# Experimental and Modelling Study of Controlled Release from Dextran-Based Cryogels

**DOI:** 10.3390/pharmaceutics16101256

**Published:** 2024-09-27

**Authors:** Carolina Lauriola, Laura Di Muzio, Patrizia Paolicelli, Maria Antonietta Casadei, Claudia Sergi, Jacopo Tirillò, Vito Cosimo Carriero, Alessandra Adrover

**Affiliations:** 1 Dipartimento di Ingegneria Chimica, Materiali e Ambiente, Sapienza Univerisità di Roma, 00184 Rome, Italy; carolina.lauriola@uniroma1.it (C.L.); claudia.sergi@uniroma1.it (C.S.); jacopo.tirillo@uniroma1.it (J.T.); 2 Dipartimento di Chimica e Tecnologia del Farmaco, Sapienza Università di Roma, 00185 Rome, Italy; laura.dimuzio@uniroma1.it (L.D.M.); patrizia.paolicelli@uniroma1.it (P.P.); mariaanonietta.casadei@uniroma1.it (M.A.C.); vitocosimo.carriero@uniroma1.it (V.C.C.)

**Keywords:** dextran-based cryogels, non-Fickian release, controlled release, cryoconcentration, transport modelling

## Abstract

In this work, five different dextran-based cryogels for controlled drug release are investigated. Vitamin B12 was used as a model drug for in vitro release tests. Two different drug-loading procedures were adopted, leading to very different drug release curves. Indeed, a fast Fickian release was observed when freeze-dried samples of DEX_40_PEG_360_MA and DEX_40_PEG_500_MA were infused with the drug after cryogel formation. On the contrary, a slowed highly non-Fickian behavior arises when the drug is loaded before the low-temperature crosslinking step, leading to the cryogel formation. The non-Fickian drug release, observed for all the five different dextran-based cryogels investigated, is actually due to the cryoconcentration phenomenon, modeled with a two-step release process. The proposed transport model accurately predicts experimental release curves characterized by a long lag time, confirming that dextran-based cryogels are suitable for controlled release.

## 1. Introduction

The use of polymeric systems for drug delivery purposes is becoming increasingly widespread [1,2,3]. Researchers focus on innovative systems that respond to the demand for cost-effectiveness, stability, simplicity and high efficacy [4,5,6]. In this regard, cryogels play a critical role [7,8,9,10,11]. In recent years, this old technology, which dates back to five decades ago, has been rediscovered due to its precious and unique properties. Cryogels share some features, such as a porous structure and superabsorbancy, with hydrogels [12,13,14,15]. Regardless, from multiple perspectives, cryogels represent an improved version of hydrogels. Specifically, they are characterized by an open interconnected macroporous structure, and consequently a higher surface area, and display optimal liquid absorption capacities and greater mechanical performance. On the contrary, hydrogels show a smaller pore size and low elasticity, which are not suitable for several applications [16,17]. The morphological and mechanical differences between hydrogels and cryogels arise from a distinct procedure of formation of the aforementioned scaffolds. As far as it concerns hydrogels, the polymerization and crosslinking process occur at room temperature. Cryogels, instead, are formed at a temperature below the freezing point of the solvent, and therefore polymerization and crosslinking occur in the interstitial spaces between the ice crystals, which are subsequently removed by freeze-drying or thawing processes. In this way, the space previously occupied by the ice crystals, which play the role of porogens, is replaced by the corresponding interconnected porous network. Although low temperatures reduce the kinetics of the polymerization and crosslinking reactions according to Arrhenius’ law, the partial freezing of the solvent determines the concentration of the polymer solution—referred to as cryoconcentration—in liquid microvolumes to the advantage of the gelation process. The choice of polymer, which can be natural or synthetic, is directly related to its field of application and influences the final texture, biodegradability and biocompatibility of the designed cryostructures. For instance, among natural polymers, the use of agarose, alginate [18,19,20], cellulose [21,22], chitosan [9,23,24,25], chondroitin [26,27], dextran [28,29,30] and gelatin [30,31,32] is a common practice. The potential to tune the physical and chemical properties of these macroporous sponge-like matrices has allowed them to be exploited for a wide variety of applications [33,34,35,36,37,38,39]. Cryogels are becoming very interesting in the pharmaceutical industry, where they can serve as carriers for the sustained release of drugs and even cells. Indeed, as reviewed in depth by Memic et al. [40], cryogels have been particularly investigated as injectable depots for local drug delivery, where release kinetics may be controlled by modifying the cryogel structure or incorporating other drug delivery modifiers into the scaffold. Therefore, a deeper understanding of the mechanism of drug release [41] from these delivery vehicles could help to optimize drug delivery systems and improve the safety and efficacy of therapies. Despite numerous studies attempting to model the release from polymeric matrices, including hydrogels [42,43,44,45,46], few articles have developed models that accurately describe the behavior of drug diffusion from cryogels [7,47,48,49,50,51]. The most applied models include the semi-empirical Higuchi and Korsmeyer–Peppas equations [52,53,54,55], which do not properly fit some experimental findings [30]. Moreover, previous studies have reported important differences in the release profiles from dextran-based cryogels and hydrogels, which were attributed to a cryoconcentration phenomenon affecting the drug during the cryogelation process [29]. Despite this effect, cryogels are not highly efficient for the sustained release of small molecules [56,57]. For this reason, different strategies have been proposed to better control the release behaviour of drugs from cryogels, including dextran-based cryogels [58]. Specifically, dextran has been combined with poly(N-isopropyl acrylamide) to develop thermoresponsive cryogels [59], with cyclodextrins [60] or with mesoporous bioactive glass [61]. Although these strategies proved to extend the drug release rate from dextran-based cryogels, better results will be achieved once the behavior of drug diffusion from cryogels can be accurately described.

In this paper, we propose a mechanistic model explaining the anomalous non-Fickian trend of release curves of vitamin B12 (cobalamin) from dextran-based cryogels. The non-Fickian behavior observed cannot be attributed to a Case II diffusion process [54,55], because the swelling process in cryogels is extremely fast and the release process occurs from a fully swollen sample. Depending on the drug-loading procedures adopted, we observed two different release behaviors: a fast Fickian release when freeze-dried samples were infused with the drug after cryogel formation, or a slowed highly non-Fickian behavior when the drug was loaded before the cryogelation process. The non-Fickian release, due to the cryoconcentration phenomenon, was modeled with a two-step release process that accurately predicts experimental release curves characterized by a long lag time and a slower release, suggesting the opportunity to exploit this attribute with a view to the controlled release of the drug. This aspect represents a determining factor for numerous therapies and several models have been proposed in the literature [62,63,64], but none of them refer explicitly to cryogels.

## 2. Materials and Methods

### 2.1. Materials

All used reagents were of analytical grade. Dextran (DEX) from *Leuconostoc mesenteroides* (Mn 40,000, DEX_40_; Mn 500,000, DEX_500_), hydroquinone mono-methyl ether, N,N′- carbonyldiimidazole (CDI), N-methyl-2-pyrrolidone, 4-dimethylaminopyridine (4-DMAP), anhydrous dimethylsulfoxide (DMSO), anhydrous tetrahydrofuran (THF), nicotinamide (NIC), ammonium persulfate (APS), N,N, N′, N′-tetramethylethylenediamine (TEMED), glycidyl methacrylate (GMA), hydroxyethyl methacrylate (HEMA), polyethylene glycol mono-methacrylate (Mn 360 and 500; PEG_360_MMA and PEG_500_MMA), deuterated water D_2_O, dialysis membranes (cut-off 12–14 kDa), methanol for HPLC (MeOH), 2-methoxyethanol and vitamin B12 were purchased from Sigma-Aldrich, St. Louis, MO, USA (Merck KGaA). Double-distilled water, absolute ethanol (EtOH), 2-methoxyethanol, acetic acid (CH_3_COOH), 37% *w*/*w* hydrochloric acid (HCl), monobasic potassium phosphate (KH_2_PO_4_) and sodium hydroxide in pellets (NaOH) were purchased from Carlo Erba.

### 2.2. Synthesis of Dextran Methacrylate

The synthesis of DEX_40_MA was carried out as reported in previous studies [30,65]. DEX_40_ (2.5 g) was solubilized in anhydrous DMSO (20 mL) at room temperature under magnetic stirring for 24 h. After complete dissolution, 4-DMAP (0.71 g) and GMA (0.26 mL) were added. The reaction mixture was maintained under magnetic stirring at room temperature for 24 h. At the end of the reaction time, the solution was precipitated dropwise into 100 mL of absolute ethanol under continuous stirring. The precipitate underwent vacuum filtration and was redissolved in distilled water (30 mL). The solution was neutralized with HCl (1N). Subsequently, it was dialyzed against distilled water for three days and finally it was freeze-dried. To characterize the lyophilised polymer, ^1^H-NMR analysis was performed with a Bruker Avance 400 spectrometer (Rheinstetten, Germany). The ^1^H-NMR spectrum was recorded in D_2_O and using NIC as the internal standard. For this purpose, 10 mg of polymer was dissolved in 0.4 mL of D_2_O and 0.2 mL of NIC solution in D_2_O (1 mg/mL). The obtained degree of methacrylation was 5 ± 1%.

### 2.3. Synthesis of Dextran Derivatives

The other methacrylic derivatives of dextran were synthesized as described in previous works [66,67], with slight modifications. The synthesis procedure involved two steps. In the first one, 2-HEMA (0.29 g, 2.2 mmol), PEG_360_MMA (0.80 g, 2.2 mmol) or PEG_500_MMA (1.10 g, 2.2 mmol) was dissolved in anhydrous THF (7 mL) and, following this, CDI (0.37 g, 2.2 mmol) was added. The reaction was carried out under a nitrogen atmosphere for 16 h at room temperature. So, hydroquinone mono-methyl ether (0.12 g) was added to the intermediate and HEMA-IC (hydroxyethyl methacrylate N-imidazoylcarbamate) or PEGMA-IC (polyethylene glycol N-imidazoylcarbamate) was obtained after evaporating THF under reduced pressure conditions. In the second step, DEX (2.4 g, 14.8 mmol of glucose repetitive unit) was dissolved in anhydrous DMSO (18 mL in the case of DEX_40_ and 25 mL in the case of DEX_500_) and the catalyst 4-DMAP (0.47 g, 3.85 mmol) was introduced. HEMA-IC or PEGMA-IC were added to the mixture without purification and the system was left to react for 24 h under magnetic stirring at room temperature. The reaction scheme of the synthesis of DEX_40_MA and dextran derivatives is reported in Supplementary Information, Figure S1A,B. The obtained mixture was precipitated dropwise in absolute ethanol (100 mL) for DEX_40_ and in methoxyethanol (150 mL) for DEX_500_. The subsequent filtration allowed the separation of the solid phase which was dissolved in distilled water (20 mL for DEX_40_ and 40 mL for DEX_500_) and neutralized with HCl (2 M) to avoid basic hydrolysis. The polymeric solution was dialyzed in distilled water, frozen and then lyophilized by using a LIO 5P freeze-dryer (5 Pascal, Milan, Italy). After the freeze-drying process, the polymers were characterized through ^1^H-NMR analysis. The ^1^H-NMR spectra were recorded in the presence of NIC used as the internal standard. ^1^H-NMR samples were prepared by solubilizing 10 mg of polymer in 0.4 mL of D_2_O and 0.2 mL of NIC solution in D_2_O (1 mg/mL). The degree of derivatization DD% was calculated based on the peaks of the standard and of the methacrylic groups. The value of the achieved DD% was of 5 ± 1% for all the synthesized polymers. For an in-depth discussion of ^1^H-NMR spectra of dextran derivatives, see [66].

### 2.4. Cryogel Preparation

Cryogels were prepared by a free radical crosslinking reaction in distilled water using the redox initiation system APS/TEMED. A certain amount of DEX_40_MA, DEX_40_HEMA, DEX_40_PEG_360_MA, DEX_40_PEG_500_MA or DEX_500_PEG_360_MA, corresponding to 0.035 mmol of methacrylic groups, was dissolved in distilled water through magnetic stirring. Following the complete solubilization of the polymer, APS (96 μL) and TEMED (54 μL) were added and after 30 s the systems were put into a cryostat (already set at the temperature of −12 °C) for two hours before undergoing the freeze-drying step. A pictorial representation of all the steps required for cryogel preparation is reported in Supplementary Information, Figure S2. The freeze-drying process was carried out with a LIO 5P bench freeze-dryer (5 Pascal, Italy) equipped with a vacuum pump Adixen (Alcatel, France). The polymer solutions were prepared in cylindrical glass molds (diameter 20 mm, height 40 mm) at room temperature. The final volume of the polymer solutions was 2 mL, which allowed us to obtain cylindrical lyophilized cryogel samples of 18.5 ± 0.5 mm diameter and 7.1 ± 0.2 mm height.

The method adopted for the preparation of cryogels is the result of an experimental campaign in which polymer concentration (6% *w*/*v*) and freezing temperature (−12 °C) were kept constant, whereas APS and TEMED concentrations were modified in order to vary the crosslinking rate of the polymer. We found out that we could obtain cryogel networks using APS concentrations in the range 1.5–13 mg/mL, and varying TEMED concentration according to APS, in order to keep the mmoles APS/mmoles TEMED ratio equal to 0.9 in all the cases. Based on these data, the cryogel systems investigated in this work were prepared by selecting an APS concentration within this range, specifically 2.4 mg/mL, which correspond to a mmoles APS/mmoles methacrylic groups ratio of 0.6. We are aware of the potential toxicity of the APS/TEMED redox initiation system and the need of cytotoxicity studies on the proprosed cryogel systems. However, it should be considered that one of the advantages of cryogels over the corresponding hydrogels is the need for smaller amounts of crosslinking reagents for network formation due to the cryoconcentration process. In light of this consideration, cryogels might be safer than the corresponding hydrogel systems. In any case, other biocompatible crosslinking methods can be applied to develop cryogel networks, such as those recently reviewed by Maiti et al. and Alavarse et al. [68,69], or safer alternatives to TEMED, such as those proposed by Pumford et al. [70]. We have not yet performed cytocompatiblity studies on these cryogels, although they are obviously possible, as sterile cryogels can be obtained by fabrication under aseptic conditions, with a standard procedure that requires that all the components of the pregel solution are sterilized separately, and then aseptically blended. This procedure, aimed at obtaining sterile cryogels, is usually adopted in all cases where final sterilization may cause substantial modification or degradation of the drug. We verified that the crosslinking reaction was completed after two hours. Indeed, we observed no differences between samples obtained by prolonging the cryogelation by up to four and six hours [30].

### 2.5. Absorption, Swelling and Degradation Studies

Absorption studies were performed with the intention of evaluating the maximum liquid uptake of the samples and the time required for complete swelling. For this purpose, phosphate buffer (PBS, pH 7.4) was preheated to the temperature of 37 °C and added dropwise to the surface of freeze-dried cryogels until a drop of PBS was observable at the bottom of the samples. The kinetics of swelling was determined by placing the samples in PBS (pH 7.4), kept in a thermostated water bath at the temperature of 37.0 ± 0.5 °C. The lyophilized specimens were left swelling until the swelling equilibrium was achieved. The water intake was tracked for 1 h. Specifically, at regular time intervals, cryogels were removed from the swelling medium and the excess of liquid was whipped off by percolation at atmospheric pressure. In this way, it was possible to weigh the samples. The swelling degree was evaluated as

(1) $$Q\left(t\right)=\frac{M_{sw}\left(t\right)}{M_{dry}}$$

where $M_{sw}\left(t\right)$ is the weight (g) of the swollen sample at the time *t* and $M_{dry}$ is the weight (g) of the dry sample. After the swelling studies, cryogels were left in PBS (pH 7.4) at 37.0 ± 0.5 °C and degradation rates were estimated. The samples were removed from the medium at variable intervals of time to measure their weights and to evaluate their weight variation. This procedure was repeated until the complete sample degradation. All tests were conducted in triplicate.

### 2.6. Morphological Characterization

The internal architecture of freeze-dried cryogels was observed via a field-emission scanning electron microscope (FE-SEM) MIRA 3 by Tescan. Since the samples were characterized by low electrical conductivity, they were coated with a thin layer of gold to prevent electronic charging and to improve image quality. The coating process was carried out in vacuum conditions (0.4 mbar) for 2 min by a sputter coater Edwards S150B applying a voltage of 1 kV and an electrical current of 40 mA to the gold electrode.

### 2.7. Release of Vitamin B12

Release studies of vitamin B12 were conducted on DEX_40_PEG_360_MA, DEX_40_PEG_500_MA, DEX_40_MA, DEX_40_HEMA and DEX_500_PEG_360_MA. A total of 2.5 mg of the drug was loaded on cryogel samples prepared with 120 mg of polymer, giving an approx. 50 (*w*/*w*) polymer/drug ratio, to ensure limited drug interference with the crosslinking process.

Two different loading procedures were implemented:

1. Loading before cryogel formation (pre-loading)
  Cryogels were loaded with 2.5 mg of vitamin B12 by introducing a solution of vitamin B12 in distilled water (2.5 mg/mL) directly into the system before the low-temperature crosslinking step, which was carried out as reported in Section 2.4, leading to the cryogel formation.
2. Loading on preformed cryogel (after loading)
  Freeze-dried cryogel samples were infused with a solution of vitamin B12. The concentration of the solution was arranged so that, on the base of absorption volumes, the amount of vitamin B12 in the cryogel was 2.5 mg. After loading, the specimens were lyophilized.

Each scaffold was placed in 20 mL of PBS (pH 7.4) under mild magnetic stirring. Temperature was maintained at 37.0 ± 0.5 °C by a water bath. At defined intervals of time, aliquots of 250 μL of the release medium were taken and immediately replaced with an identical volume of fresh PBS, so that the reservoir volume (20 mL) remained constant. Withdrawals were made every minute for the first 5 min, then every 5 min up to 20 min, every 10 min until the first hour passed, every 30 min up to 2 h and then every hour up to 8 h. The last two aliquots were withdrawn after 24 and 48 h. The concentration $c_{res}$ of the released drug in the reservoir volume was evaluated by HPLC analysis. The HPLC consisted of a Perkin Elmer Series 200 LC pump, supplemented with a 235 Diode Array Detector and equipped with a Merck Hibar LiChrocart (250-4, 5 μm) RP-18 column. A 65:35 mixture of 0.01 M CH_3_COOH/MeOH was used as the eluent. The flow rate was set to 0.7 mL/min. Aliquots of 25 μL of every sample solution were injected into the HPLC system. A calibration curve c[mg/mL] = 0.0465A[V × s], valid in the concentration range c ∈ [0.0005, 0.16] mg/mL and reported in Supplementary Information Figure S3, was constructed to quantify the concentration of vitamin B12 in the reservoir at each withdrawal time instant ($\lambda_{max}$ = 361 nm). All experiments were conducted in triplicate. Release profiles were reported as $c_{res}$ vs. *t* and $M_{t}/M_{\infty }$ vs. time *t* [min] (amount of drug released up to time *t* rescaled onto the total amount of drug released after an infinite time) with the corresponding standard deviations.

## 3. Results and Discussion

### 3.1. Swelling and SEM Analysis

**Figure 1:**
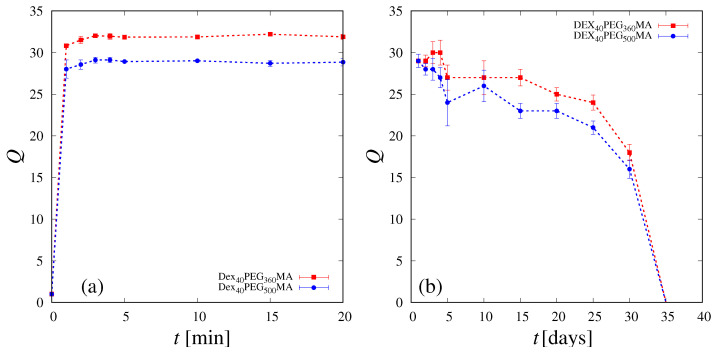
Swelling degree curves (**a**) and degradation curves (**b**) of freeze-dried DEX_40_PEG_360_MA cryogels and DEX_40_PEG_500_MA cryogels.

**Figure 2:**
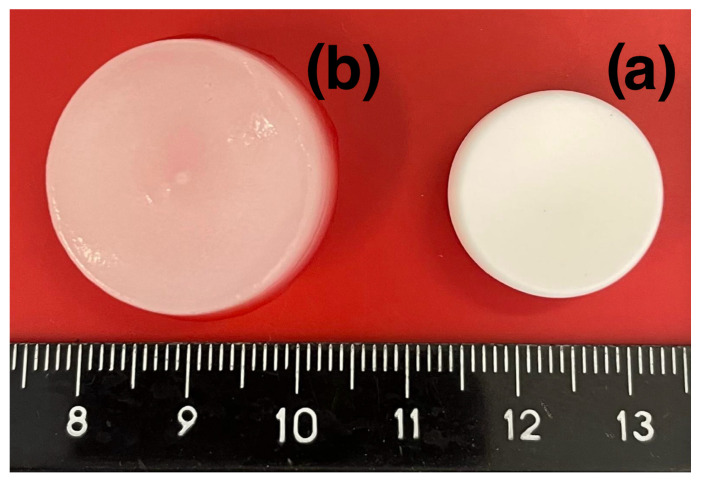
Freeze-dried DEX_40_PEG_360_MA cryogel before (**a**) and after complete swelling (**b**).

Figure 1a shows the swelling degree Q(t) of the DEX_40_PEG_360_MA and DEX_40_PEG_500_MA cryogels as a function of time *t*. Almost complete swelling is attained within 2 min for both cryogels, which also share a comparable degradation time, as shown in Figure 1b. The swelling data are reported in Figure 1a for the first 20 min in order to better show the rapid saturation on the asymptotic values which remain essentially unchanged until the end of the experiment (1 h). Figure 2 shows a picture of a DEX_40_PEG_360_MA cryogel before (a) and after complete swelling (b).The degradation data shown in Figure 1b have larger errors than swelling data (as highlighted by the error bars) intrinsically due to the experimental procedure, but show a well-defined decaying behavior, with a total degradation time of 35 days for both cryogels.

**Figure 3:**
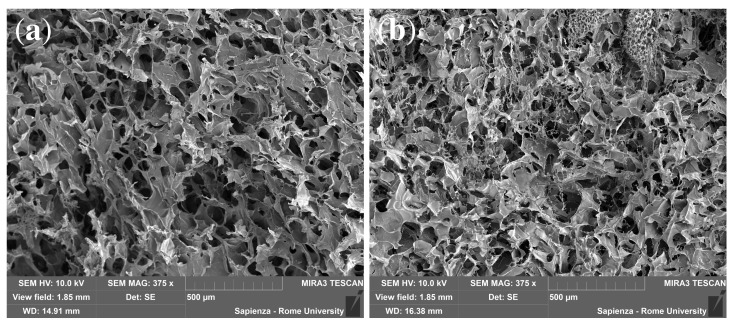
SEM images showing the morphology of cryogels. (**a**) DEX_40_PEG_360_MA. (**b**) DEX_40_PEG_500_MA.

It can be observed that a slightly lower equilibrium swelling degree is observable for DEX_40_PEG_500_MA. It can be attributed to the formation of clusters between the oxyethylene units, resulting in a greater degree of interaction between the polymer chains and the formation of conglomerates which, in turn, determine the smaller mesh of the polymer network and a lower capability of solvent penetration, as shown by SEM analysis in Figure 3a,b. Indeed, during freeze concentration, the dextran/PEG concentrations might become high enough for phase separation to occur, thus forming dextran and PEG clusters, as observed by Heller et al. [71].

### 3.2. Release Studies

**Figure 4:**
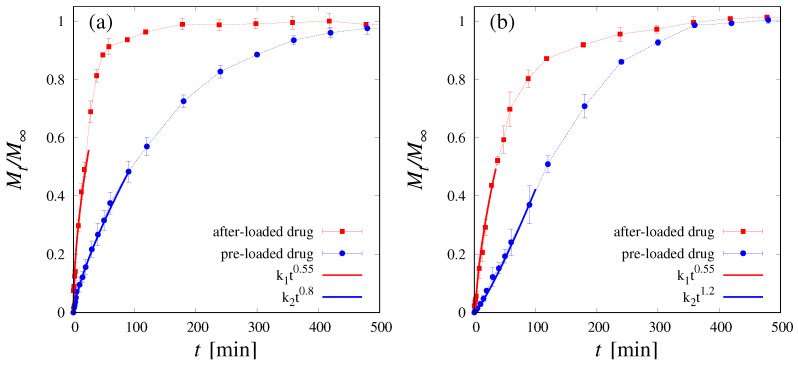
Release curves of vitamin B12 in the case of pre-loaded drug (blue curves) and after-loaded drug (red curves). (**a**) Release from DEX_40_PEG_360_MA cryogel. (**b**) Release from DEX_40_PEG_500_MA cryogel.

Release curves, shown in Figure 4a for DEX_40_PEG_360_MA and in Figure 4b for DEX_40_PEG_500_MA, are highly sensitive to the drug-loading procedure. Specifically, for both cryogels analyzed, the release curve for the after-loaded drug is significantly faster than that obtained with the pre-loaded drug. Moreover, the after-loaded release curve exhibits, for both cryogels, a well-defined power-law behavior $M_{t}/M_{\infty }∼t^{n}$ at a short–intermediate time-scale, characterized by an exponent n=0.55 close to n=0.5 characterizing the Fickian behavior [54]. On the contrary, the pre-loaded release curves exhibit a markedly non-Fickian behavior characterized by an exponent *n* significantly larger than 0.5; specifically, n=0.8 for DEX_40_PEG_360_MA and n=1.2 for DEX_40_PEG_500_MA. The larger the value of the *n* exponent, the longer the lag time observed in the release curves [64].

A similar non-Fickian behavior was already observed for methacryloyl gelatin (GelMA) blended with dextran methacrylate (DEX_40_MA) cryogels [30] and explained as a consequence of the cryoconcentration phenomenon. Indeed, the cryogel can be viewed as a hierarchical porous structure characterized by the coexistence of two phases, a microporous dense stationary one, in which the drug (in the pre-loaded case) gets trapped during the cryogelation process, and a macroporous “mobile” one. During the release process, the cryogel sample rapidly swells, and the drug, initially trapped in the microporous phase, is irreversibly released in the macroporous phase where it is free to diffuse out of the sample. Therefore, the release process in the pre-loading case occurs in two steps characterized by two different time-scales: the time-scale $t_{t}$, associated with the internal drug-transfer process from the stationary to the mobile phase (controlling the lag time), and the time-scale $t_{d}$, associated with diffusive transport in the macroporous phase. The interplay between these two transport processes is responsible for the observed non-Fickian behavior of the release curves if the drug is pre-loaded. On the contrary, if the drug is infused in the lyophilized sample after the cryogel formation (after-loading procedure), there is no drug trapping in the microporous phase (absence of the cryoconcentration phenomenon) and drug release occurs exclusively by diffusive transport in the macroporous phase. By considering that the swelling process is extremely fast, compared to the diffusional time-scale, the release curves exhibit a typical Fickian behavior, as experimentally observed.

In the presence of cryoconcentration, the more pronounced non-Fickian behavior of DEX_40_PEG_500_MA with respect to DEX_40_PEG_360_MA is due to a larger fraction of the drug initially being trapped in the microporous phase. This finding is in agreement with the reduced swelling capability of DEX_40_PEG_500_MA due to the formation of clusters between the oxyethylene units, resulting in a greater degree of interaction between the polymer chains and the formation of conglomerates, as shown by SEM images.

### 3.3. Theoretical Model of Drug Release in the Pre-Loading Case

The transport model developed is based on the coexistence of two phases: a microporous stationary phase, in which the vitamin is initially concentrated due to the cryoconcentration phenomenon, and a macroporous mobile phase where drug transport occurs exclusively by diffusion. Indeed, the swelling process of the lyophilized sample is extremely fast (in the order of 2 min to reach equilibrium conditions) so that the cryogel is assumed to be fully swollen at the beginning of the release process. For this reason, the non-Fickian release observed cannot be attributed to a Case II transport process [54,55], in which drug release and swelling are two phenomena occurring simultaneously. When $R_{sw}≃13$ mm and $H_{sw}≃10$ mm are the dimensions (radius and height) of the swollen cylindrical sample (volume $V_{cyl}=\pi R_{sw}^{2}H_{sw}$) and $m_{0}≃2.5\pm 0.1$ mg is the amount of drug loaded in the cryogel, the initial drug concentration in the specimen is $c_{0}=m_{0}/V_{cyl}≃0.47$ mg/mL.

The drug trapped in the microporous phase, with the initial concentration $c_{s}\left(r,z,0\right)=\varepsilon_{0}c_{0}$, is irreversibly released in the macroporous mobile phase with a linear transfer rate $k_{t}c_{s}$, directly proportional to the local drug concentration $c_{s}\left(r,z,t\right)$. The fraction $\varepsilon_{0}$ of drug initially trapped in the microporous phase is one important parameter of the model as well as the transfer coefficient $k_{t}\left[s^{-1}\right]$ controlling the time-scale $t_{t}=1/k_{t}$ associated with the internal drug-transfer process between phases. Once in the mobile phase, the drug is free to diffuse out of the sample into the liquid reservoir (volume $V_{res}$) where perfect mixing conditions are ensured by agitation.

Let $c_{m}\left(r,z,t\right)$ and $c_{res}\left(t\right)$ be the drug concentrations in the mobile phase and in the reservoir, respectively, with the initial conditions $c_{m}\left(r,z,0\right)=\left(1-\varepsilon_{0}\right)c_{0}$ and $c_{res}=0$. The coupled balance equations for $c_{s}$, $c_{m}$ and $c_{res}$ read as

(2) $$\begin{array}{ccc} \frac{\partial c_{s}\left(r,z,t\right)}{\partial t} & = & -k_{t}c_{s} \end{array}$$

(3) $$\begin{array}{ccc} \frac{\partial c_{m}\left(r,z,t\right)}{\partial t} & = & D_{B12}\nabla^{2}c_{m}+k_{t}c_{s} \end{array}$$

(4) $$c_{m}\left(r,z,t\right){|}_{S}=c_{res}\left(t\right),\;\frac{\partial c_{m}}{\partial r}{|}_{r=0}=0$$

(5) $$\begin{array}{ccc} V_{res}\frac{dc_{res}\left(t\right)}{dt} & = & D_{B12}\int_{S}-\nabla c_{m}\cdot n\;ds-\sum_{i=1}^{N_{w}^{t}}V_{w}c_{res}\delta \left(t-t_{w}^{i}\right) \end{array}$$

where δ(t) is a Dirac delta function, $\nabla \left(\cdot \right)=\left(\frac{\partial \cdot }{\partial r},\frac{\partial \cdot }{\partial z}\right)$ and $\nabla^{2}\left(\cdot \right)=\frac{\partial^{2}\cdot }{\partial z^{2}}+\frac{1}{r}\frac{\partial }{\partial r}\left(r\frac{\partial \cdot }{\partial r}\right)$ are the gradient and Laplacian operators in cylindrical coordinates, *S* is the lateral surface of the cylindrical specimen, n is the outward unit normal vector of *S* and $D_{B12}$ is the diffusion coefficient of B12 in PBS in the macroporous phase.

Numerical results for the concentration fields were obtained by solving the transport scheme with a commercial software enforcing the Finite-Element Method (Comsol 5.5). For the numerical integration of the coupled time-dependent transport equations of $c_{s}$ and $c_{m}$, the Coefficient Form PDE package was adopted for the 2-d axial symmetric geometry (cylinder) and solved in transient conditions (time-dependent solver, fully coupled method) together with the ODE for $c_{\mathrm{res}}$. Lagrangian quadratic elements were chosen. The MUMPS solver was adopted with the relative tolerance $10^{-6}$.

The time-dependent Dirichlet boundary condition, Equation (4), replaces the generally adopted perfect sink condition ($c_{m}{|}_{S}=0$) that is not appropriate for describing our experiments in which the ratio α

(6) $$\alpha =V_{cyl}/V_{res}$$

is about 1/4 and therefore is not small enough for the perfect sink approximation to be valid (α≪1). Increasing the volume of the reservoir is to be avoided, as the concentration of the drug, at the first withdrawals, would be too low and also would worsen the mixing conditions in the reservoir.

The adoption of Equation (4) for $c_{m}$ at the boundary *S* requires the introduction of the balance Equation (5) for $c_{res}$. Equation (5) takes into account the finite volume of the reservoir and the $N_{w}$ withdrawals (each with volume $V_{w}$) that are made at different time instants ${\left\{t_{w}^{i}\right\}}_{i=1}^{N_{w}}$ in order to measure, by HPLC, the drug concentration in the reservoir $c_{res}\left(t_{i}\right)$ at the withdrawal time $t_{i}$ and build up the experimental $M_{t}\left(t_{i}\right)$ vs. $t_{i}$ release curve

(7) $$M_{t}\left(t_{i}\right)=c_{res}\left(t_{i}\right)V_{res}+\sum_{j=1}^{N_{w}^{t}}c_{res}\left(t_{j}\right)V_{w}$$

where $N_{w}^{t}$ denotes the number of withdrawals made from time t=0 to time *t* (or equivalently $t_{i}$). Equation (7) can be also adopted to evaluate the amount of drug $M_{t}\left(t\right)$ released up to time *t* from the transport model Equations (2)–(5), by simply replacing $c_{res}\left(t_{i}\right)$ with $c_{res}\left(t\right)$.

The finite value of α and the withdrawals influence the total amount of drug $M_{\infty }$ that can be asymptotically released [72,73]. Indeed, according to Equation (7), $M_{\infty }$ attains the form

(8) $$M_{\infty }=c_{res}^{\infty }V_{res}+\sum_{j=1}^{N_{w}}c_{res}\left(t_{j}\right)V_{w}=\frac{c_{0}V_{cyl}}{1+\alpha }+\frac{\alpha }{1+\alpha }\sum_{j=1}^{N_{w}}c_{res}\left(t_{j}\right)V_{w}$$

where $c_{res}^{\infty }=\left(c_{0}V_{cyl}-\sum_{j=1}^{N_{w}^{t}}c_{res}\left(t_{j}\right)V_{w}\right)/\left(V_{cyl}+V_{res}\right)$ is the asymptotic concentration in the reservoir, coinciding with the uniform drug concentration $c_{m}^{\infty }$ that asymptotically sets in the mobile phase when all components of the concentration gradient nihilate ($\nabla c_{m}=0$), and the drug release from the specimen to the reservoir stops. From Equation (8), it can be readily observed that, the larger is the value of α, the more $M_{\infty }$ deviates from (and is smaller than) $m_{0}=c_{0}V_{cyl}$. On the contrary, the effect of the withdrawals is to increase the amount of drug asymptotically released.

Given the large porosity of the cryogels and the relatively small molecular weight of B12, the diffusion coefficient $D_{B12}$ can be assumed to be close to the bare diffusion coefficient of B12 in PBS. Specifically, we assumed $D_{B12}=7.2\times 10^{-10}$ m^2^/s for both the DEX_40_PEG_360_MA and DEX_40_PEG_500_MA cryogels. See Section 3.4 for a detailed analysis of the physical/mathematical considerations supporting this choice.

The two parameters to tune in order to fit the experimental data are the fraction $\varepsilon_{0}$ of B12 initially trapped in the microporous phase and the transfer rate coefficient $k_{t}$. The best-fit value for $k_{t}$ is $k_{t}=2.6\times 10^{-4}$ s^−1^ for both cryogels. The two cryogels differ in the amount of vitamin initially trapped in the microphase, namely $\varepsilon_{0}=0.80$ for DEX_40_PEG_360_MA and $\varepsilon_{0}=0.95$ for DEX_40_PEG_500_MA. This difference, as in the case of the equilibrium swelling degree, could be explained by the larger amount of conglomerates in the DEX_40_PEG_500_MA due to a stronger interaction between the chains. This results in a smaller mesh size of the polymer network, which then traps a larger fraction of the drug.

**Figure 5:**
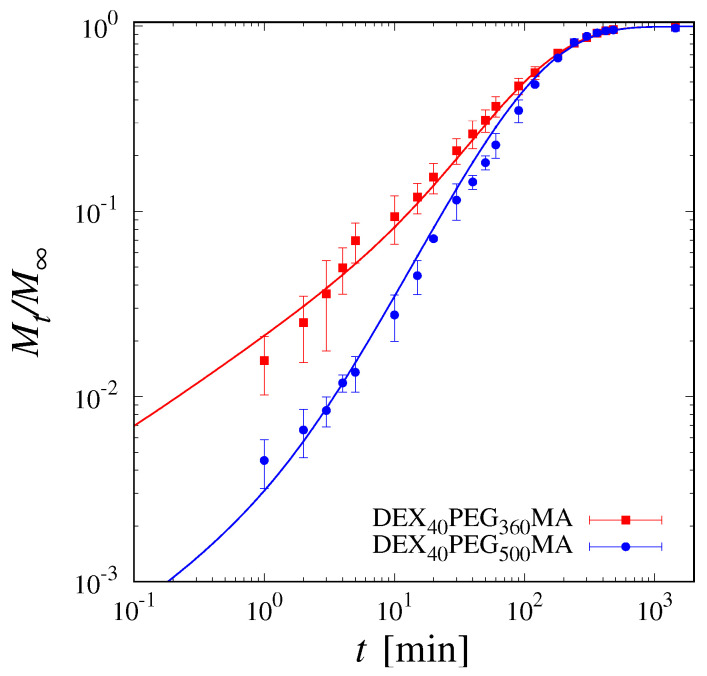
Log−log plot of release data of vitamin B12 (pre-loaded) from DEX_40_PEG_360_MA and DEX_40_PEG_500_MA cryogels. Continuous lines indicate model predictions.

The excellent agreement between model predictions and experimental release curves for DEX_40_PEG_360_MA and DEX_40_PEG_500_MA in the pre-loaded case is shown in Figure 5.

**Figure 6:**
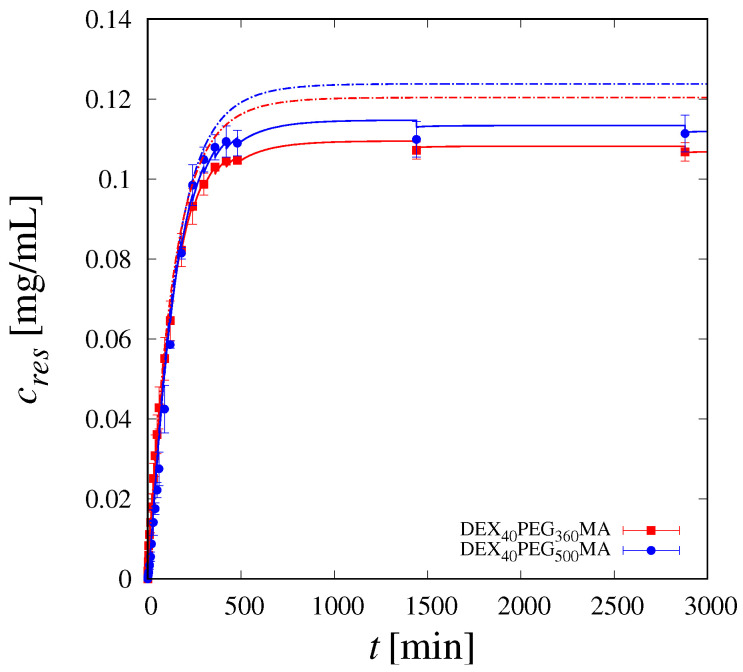
Comparison between experimental results for $c_{res}\left(t_{i}\right)$ (points) and model predictions. Continuous lines are numerical results accounting for the effect of withdrawals on release dynamics. Dashed lines are numerical results neglecting the effect of withdrawals on release dynamics.

Figure 5 clearly shows how the model is capable of describing the lag time and the non-Fickian behaviour of the release curves, as well as the long-term saturation towards the complete release. As a further confirmation, Figure 6 shows a quantitative comparison between the experimental values of $c_{res}$ at different withdrawal time instants $t_{i}$ and model predictions.

Figure 6 allows us to appreciate the importance of taking into account the effect of withdrawals on release dynamics [72,73]. Indeed, each withdrawal (and subsequent replenishment of the same aliquot with fresh solution) results in an immediate decrease in the drug concentration in the reservoir, and this affects the release dynamics, especially in the case of frequent withdrawals on intermediate time-scales. Actually, Figure 6 shows the excellent agreement between the experimental data and model predictions when withdrawals are properly accounted for (continuous lines). On the contrary, a significant deviation between the experimental data and model predictions (dashed lines) can be observed if withdrawals are neglected in the dynamics of $c_{res}$. Neglecting withdrawals in modeling the release dynamics is possible, but this may lead to significant errors in the estimate of transport parameters, namely $k_{t}$ and $\varepsilon_{0}$.

Finally, it is worth noting that the best-fit values of the transfer rate coefficient $k_{t}$ implies a value of the Damkölher number Da in the order of ten. Specifically,

(9) $$Da=\frac{t_{d}}{t_{t}}=\frac{\frac{{\left(H_{sw}/2\right)}^{2}}{D_{m}}}{\frac{1}{k_{t}}}=\frac{k_{t}{\left(H_{sw}/2\right)}^{2}}{D_{B12}}≃9$$

By considering that Da represents the ratio between the characteristic diffusion time $t_{d}$ and the characteristic transfer time $t_{t}$, a value of Da significantly larger than unity implies that the internal drug transfer between the micro- and the macrophase is a fast process, controlling the temporal evolution of the release curves at short/intermediate time-scales. On the contrary, the long-term behavior is controlled by drug diffusion in the macroporous phase, and this allows us to estimate the value of the diffusion coefficient $D_{B12}$, as discussed in detail in Section 3.4.

**Figure 7:**
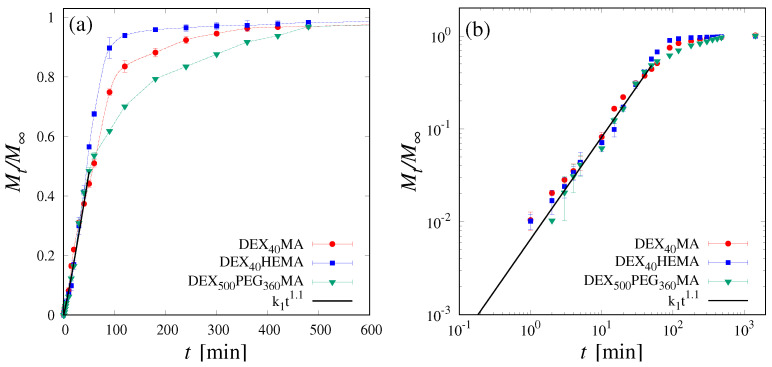
(**a**,**b**) $M_{t}/M_{\infty }$ vs. *t* for pre-loaded B12 from DEX_40_MA, DEX_40_HEMA and DEX_500_PEG_360_MA cryogels. The continuous black line highlights the non-Fickian behavior $M_{t}/M_{\infty }∼t^{n}$ with n=1.1.

The interplay between internal transfer and diffusion in the mobile phase, at short intermediate time-scales, is responsible for the non-Fickian behaviour, more pronounced for DEX_40_PEG_500_MA because a larger fraction of drug is initially trapped in the stationary phase. The non-Fickian behavior of the release curve is a common feature of many dextran-based cryogels when the drug is loaded before the cryogelation process. Indeed, Figure 7 shows the release curves $M_{t}/M_{\infty }$ vs. *t* of pre-loaded B12 for DEX_40_MA, DEX_40_HEMA and DEX_500_PEG_360_MA. The corresponding raw release data $c_{res}$ vs. *t* are shown in Supplementary Information, Figure S4.

The log–log plot allows us to highlight the non-Fickian behavior characterized by an exponent n=1.1 for all these dextran-based cryogels. The B12 release is characterized by the same lag time for all the cryogels, and represents the fingerprint of drug trapping in the stationary phase induced by the cryoconcentration process.

### 3.4. Estimation of the Diffusion Coefficient $D_{B12}$

On a long time-scale, when the release process is controlled solely by drug diffusion in the macroporous phase, the fraction of unreleased drug $1-M_{t}/M_{\infty }$ in the cylindrical sample decays exponentially over time *t* as

(10) $$1-\frac{M_{t}}{M_{\infty }}∼e^{-D_{B12}\;t\lambda_{0}^{2}},\;\lambda_{0}^{2}=\alpha_{1}^{2}+\frac{\pi^{2}}{H_{sw}^{2}}$$

where $\lambda_{0}^{2}$ is the first (dominant) eigenvalue of the Laplacian operator $\nabla^{2}$ in cylindrical coordinates with homogeneous Dirichlet boundary conditions and $\alpha_{1}$ is the first positive root of the $J_{0}$ Bessel function $J_{0}\left(\alpha_{1}R_{sw}\right)=0$. For the dimensions $R_{sw}$ and $H_{sw}$ of the swollen samples under investigation, the dominant eigenvalue $\lambda_{0}$ is equal to $\lambda_{0}=13.571\times 10^{4}$ [m^−2^].

**Figure 8:**
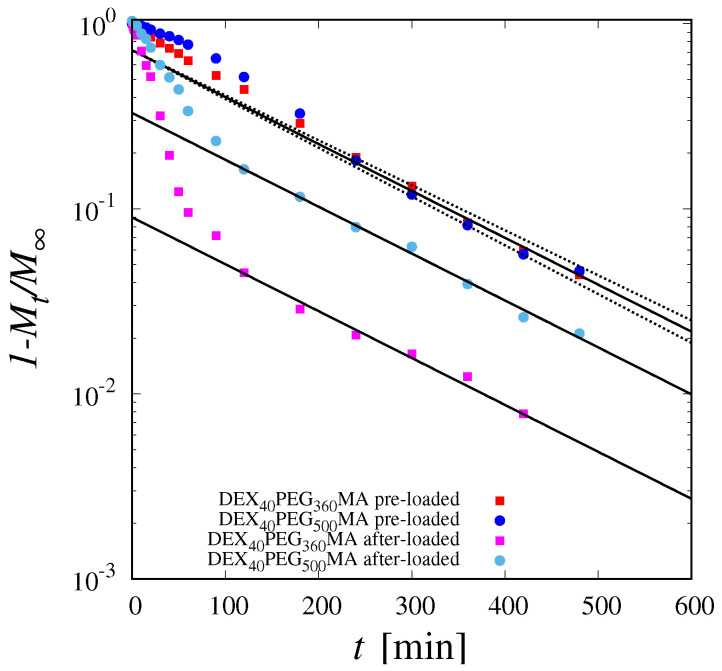
Log−normal plot of $1-\frac{M_{t}}{M_{\infty }}$ vs. *t* for DEX_40_PEG_360_MA and DEX_40_PEG_500_MA for both drug loading conditions (before and after cryogel formation). Continuous lines indicate the exponential decay, Equation (10), with $D_{B12}=\left(7.17\pm 0.3\right)\times 10^{-10}$ m^2^/s. Dashed lines highlight the confidence interval.

Figure 8 shows the decay of $1-M_{t}/M_{\infty }$ vs. *t* for both cryogels DEX_40_PEG_360_MA and DEX_40_PEG_500_MA and for both loading conditions (before and after cryogel formation). The log-normal scale adopted highlights that, on long time-scales, the trend is indeed an exponential decay over time, characterized by the same exponent for all the curves. By best-fitting the experimental exponential decay of $1-M_{t}/M_{\infty }$ vs. *t* with Equation (10), it is possible to estimate the diffusion coefficient $D_{B12}$ in the macroporous phase, thus obtaining $D_{B12}=\left(7.17\pm 0.3\right)\times 10^{-10}$ m^2^/s. The long-term exponential decay is the same for all the curves, whether the drug was loaded before cryogel formation or after cryogel formation by infusion. This confirms that the lag effect observed in the release curves in the case of pre-loaded drugs is due solely to the entrapment of the drug in the stationary phase by cryoconcentration.

## 4. Conclusions

This study demonstrates the potential of dextran-based cryogels for controlled drug release applications, using vitamin B12 as a model drug for in vitro release tests. The research highlights how different drug loading procedures significantly influence the release kinetics. Specifically, freeze-dried samples infused with the drug after cryogel formation exhibit a fast Fickian release. In contrast, when the drug is loaded before the low-temperature crosslinking step that leads to cryogel formation, a slowed, highly non-Fickian release behavior is observed. This non-Fickian release is attributed to the cryoconcentration phenomenon and is accurately modeled using a two-step release process. The mechanistic model proposed in this study successfully explains the anomalous non-Fickian release trends, distinguishing it from other diffusion processes such as Case II diffusion. This study highlights the importance of the drug loading procedure in tailoring drug release profiles from cryogels, offering valuable insights for the development of controlled-release therapies. The cryogels proposed in this work may also be suitable for drug molecules of higher molecular weight. Indeed, we have already reported on the possibility of applying these dextran derivatives for the development of injectable and in situ gelling drug delivery systems, specifically designed for the controlled release of large molecules [66]. Certainly, the carbonate ester makes these polymer networks easily degradable in vivo. Therefore, these dextran derivatives can certainly also be proposed for the delivery of high-molecular-weight drugs with cryogel systems. 

## Data Availability

Data are available from the corresponding author upon request.

## Supplementary Materials

The following supporting information can be downloaded at: https://www.mdpi.com/article/10.3390/pharmaceutics16101256/s1, Figure S1: Scheme of the synthesis of DEX_40_MA (A) and the other dextran derivatives, i.e., DexHEMA and DexPEGMA (B); Figure S2: Pictorial representation of all the steps required for cryogel preparation; Figure S3: HPLC calibration curve of Vitamin B12; Figure S4: Raw release data c_*res*_ vs. t for DEX_40_MA, DEX_40_HEMA and DEX_40_PEG_40_MA
