# Supplementary material for: Experimental and Modelling Study of Controlled Release from Dextran-Based Cryogels
Source: Pharmaceutics. 2024 Sep 27;16(10):1256. doi: 10.3390/pharmaceutics16101256 (PMC11510673; doi:10.3390/pharmaceutics16101256)
Supplement: Supplementary file 1 [file pharmaceutics-16-01256-s001.zip › pharmaceutics-3213953-supplementary.pdf]

Supplementary Information

Experimental and modelling study of controlled release from Dextran-based cryogels

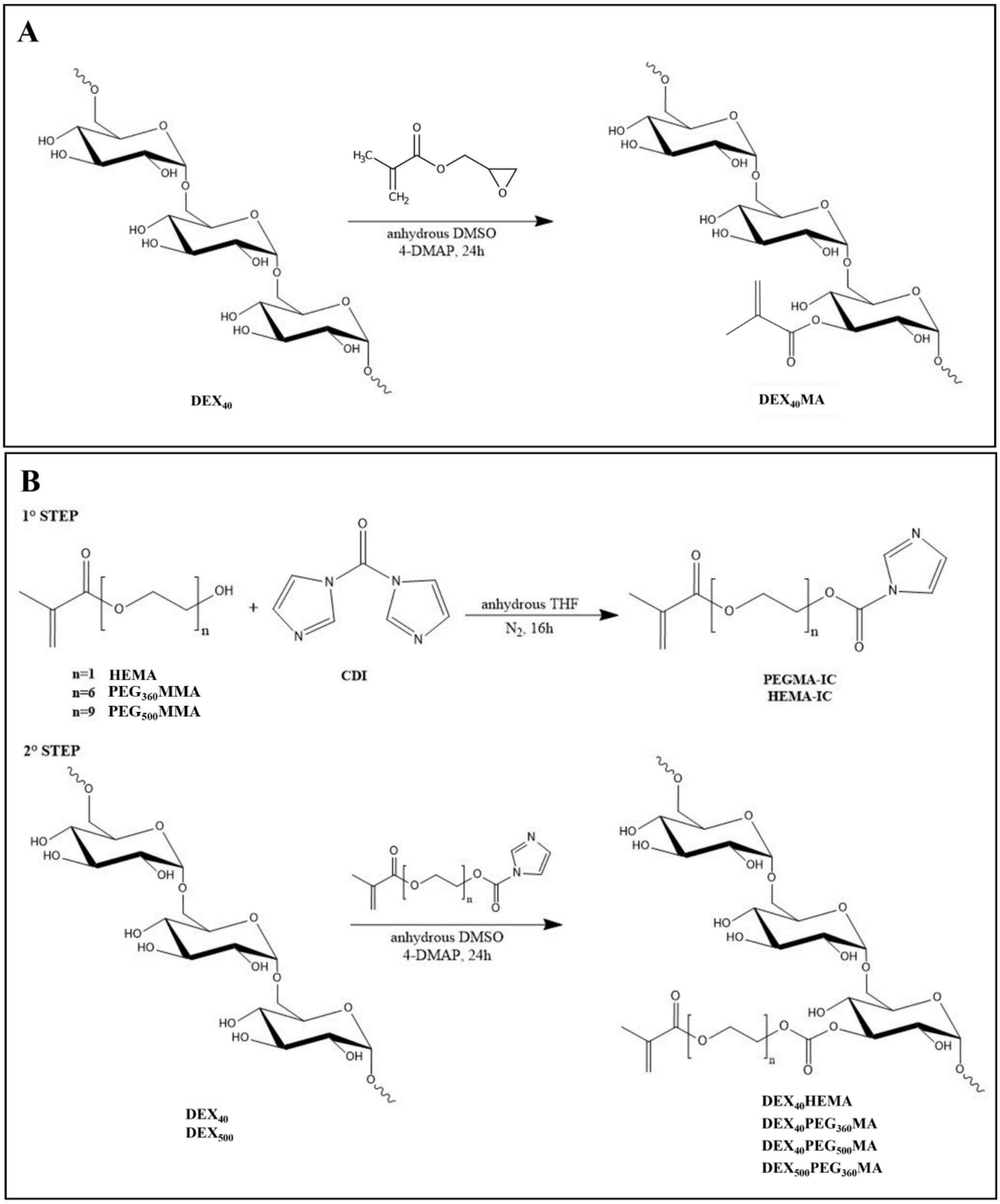

Figure S1: Scheme of the synthesis of $DEX_{40}MA$ (A) and the other dextran derivatives, i.e. DexHEMA and DexPEGMA (B).

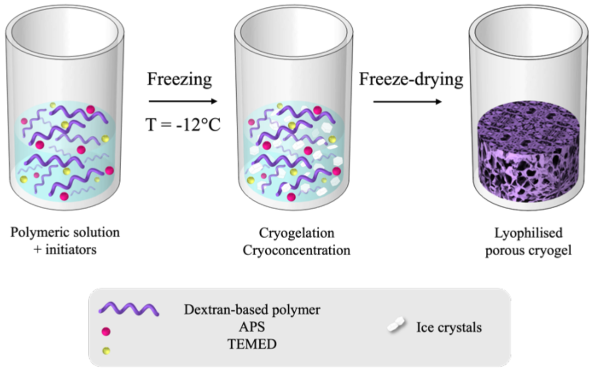

Figure S2: Pictorial representation of all the steps required for cryogel preparation.

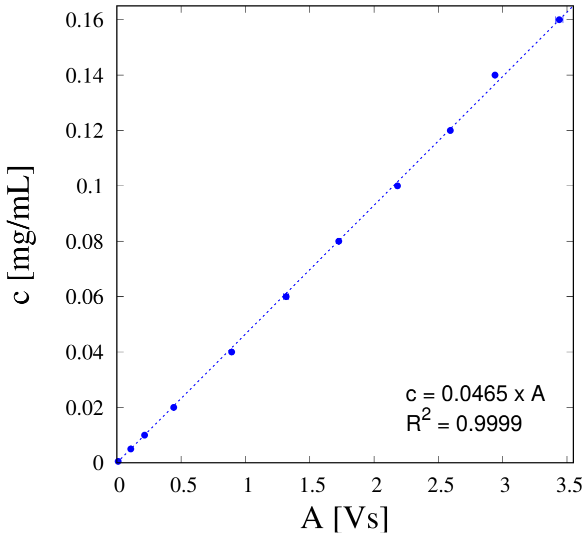

Figure S3: HPLC calibration curve of Vitamin B12

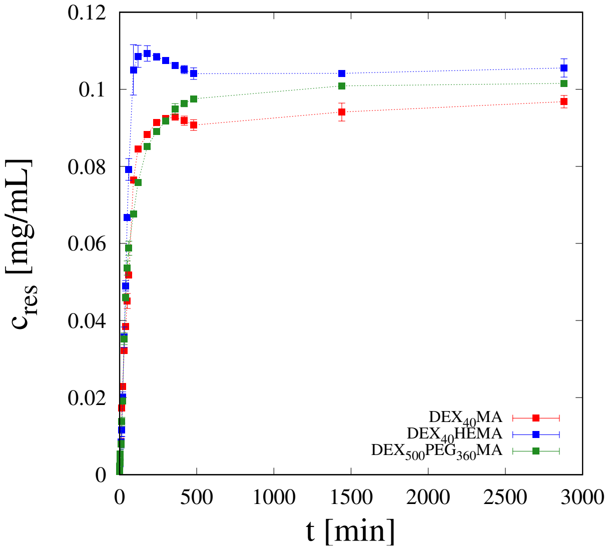

Figure S4: Raw release data c_{res} vs t for $DEX_{40}MA$, $DEX_{40}HEMA$ and $DEX_{500}PEG_{360}MA$
